# Meeting of the “Faculties” at Washington, D. C.

**Published:** 1904-07

**Authors:** 


### MEETING OF THE "FACULTIES" AT WASHINGTON, D. C.

The meeting of this organization at Washington, D. C., June 9 to 11, 1904, must be regarded as the most important held in recent years by that body. While the one which decided to advance the dental course to four years marked an epoch in dental education, it did not compare, in point of interest, to the one that has just closed its sessions.

It was generally understood that a determined effort would be made to return to three years and six or seven months, and those who attended the meeting were not disappointed. The subject was the main topic that occupied the thoughts of the members; in fact, but little else was considered of moment in comparison. The members discussed it in meetings and in the hotel corridors. The result was at all times in doubt, for it was plainly evident that a very large number of the dental colleges of the country were earnestly in opposition to a further continuance of the four years' course.

It was the general opinion that the question, in all its length and breadth, had been discussed as no other question had been in the past at meetings of this organization. Upon both sides there had been exhibited an earnest effort to meet the problems connected with the question with the dignity due to its importance and to the interests involved. It was very evident that the motive for a retrograde action was mainly one of finance. The large majority of the schools desiring a change were being seriously affected by the four years' course; indeed, with some it had become a matter of life and death. This was fully appreciated by those in opposition to any change, and there was felt, and openly expressed, much sympathy for those who had tried the lengthened course and had severely suffered by the experiment. Especially was this felt for the Southern schools. Notwithstanding this sympathy, the determination to maintain the higher standard was not in the least lessened.

The anxiety to know how the Association stood on the question resulted in the adoption of a motion to take an informal vote upon it, but this was to be considered simply in the light of a test. The result was a surprise to both sides, as it demonstrated that the section in the Association advocating a return to three years must increase the vote if there was to be any change. This informal vote stood 19 to 27. The hope that this margin might be lessened if not entirely obliterated, by earnest efforts caused a caucus to be held and every effort made to influence those in doubt. The majority was not very hopeful of final success, and hence the interest and anxiety on both sides were intense, and were not removed until the final vote was announced and the question decided by 24 in opposition to change, to 21 in favor, and 2 not voting. This was not a very reliable majority, but it was sufficient, and the tension was greatly relieved.

Before the final adjournment, upon motion of Dr. Black, it was decided, by a vote of colleges, to give the weaker schools some relief, and the final vote was cast for four years with a six months' course. This was not altogether satisfactory to several of the colleges, for these desired three years with a nine months' course, and one or two openly stated that their schools would probably be forced out of the Association.

The final vote does not influence those departments of universities that have persistently held to four years with a nine months' course. These will continue to uphold the higher standard.

It is thought, by those best qualified to judge, that the cutting down of the matriculating list in the Freshman year will be but temporary, and that in a year or two an equilibrium will be established and the classes will resume the normal number. If this should prove not to be the case, the question may again, in the near future, cause a repetition of the effort to reduce the time, but for the present it must remain practically without alteration.

The Association made another important move in the direction of more liberal treatment of those applying for advanced standing. The "Count System" was adopted, which will permit dental colleges to place a certain valuation upon studies taken in other than strictly dental colleges. This will greatly simplify the question of entrance and do away with many unjust decisions, forced upon colleges in deciding applications for advanced standing. This decision does not apply to, nor does it affect, the preliminary entrance standards already in force.

The vote in detail upon the question will be found upon another page, and it will bear careful inspection and will doubtless be of interest to all dental educational circles in this country.

