# Dr. Black’s Correction

**Published:** 1904-08

**Authors:** G. V. Black


### DR. BLACK'S CORRECTION.

*To the Editor:*

Sir,—In your editorial on the "Meeting of the Faculties at Washington, D. C.," on page 548, in the last paragraph, you say, "Upon motion of Dr. Black, it was decided, by a vote of colleges, to give the weaker schools some relief, and the final vote was cast for four years with a six months' course." I wish to say that you are mistaken in attributing that motion to me. I not only did not make the motion, but I opposed it as sharply as I knew how at the time the motion was made and voted against it. I would be very unwilling that this go uncorrected. The whole outcome of the meeting was extremely unsatisfactory to me. The vote by which the four-year course was maintained was too small, and I felt so at the time, but I was opposed to a course of six months in any case or for any purpose.

Very truly,

G. V. Black.

[Remarks.—It is with regret that the writer failed to understand whence the motion originated to make the time four years with a course of six months. The "Foster Resolution," covering the same ground, had been under consideration, but the question had become much confused, and it was supposed the meeting was acting upon a new motion and that Dr. Black was its originator. He gave the impression to many that he favored such a change, and it seemed to the writer that this was a very creditable course to take, hence there was no hesitation in making use of his name. Dr. Black's repudiation of the statement made in our last issue, and also his opposition to the change adopted, came as a surprise, but he certainly ought to know his true position, and we, therefore, very willingly make the necessary correction.—Ed.]

